# Effects of Mindfulness-Based Cognitive Therapy on Parental Mental Health and Child Behavior in Families of Children with Autism Spectrum Disorder: A Randomized Controlled Trial

**DOI:** 10.3390/children13010053

**Published:** 2025-12-30

**Authors:** Dimitrios Papadopoulos, Katerina Maniadaki

**Affiliations:** 1 Department of Social Work, University of West Attica, 12244 Athens, Greece; maniadaki@uniwa.gr; 2 Department of Early Years Learning and Care, University of Ioannina, 45500 Ioannina, Greece

**Keywords:** Mindfulness-Based Cognitive Therapy (MBCT), mindful parenting, Autism Spectrum Disorder (ASD), parental mental health, parenting stress, child behavior, family, development, psychopathology

## Abstract

**Highlights:**

**What are the main findings?**

- Mindfulness-Based Cognitive Therapy (MBCT) adapted with mindful parenting components produced substantial reductions in parental depression, anxiety, and stress, alongside increases in positive affect and life satisfaction at post-intervention and at one month follow-up compared with a waitlist control group.
- Parents who received MBCT reported parent perceived reductions in the severity of their children’s behavior problems at post-treatment and at one-month follow-up, despite children receiving no direct intervention.

**What are the implications of the main findings?**

- Integrating MBCT with mindful parenting principles in both group and individual practice represents a feasible and acceptable family-centered approach for enhancing the mental health of parents raising a child with ASD and for supporting children’s behavioral adjustment.
- Early screening for parental depression, anxiety, and stress should be integrated into ASD services, to facilitate timely, practical, and cost-effective mental health interventions.

**Abstract:**

**Background/Objectives**: Caring for a child with Autism Spectrum Disorder (ASD) is often associated with elevated psychological distress and reduced life satisfaction. Mindfulness-based interventions may offer substantial benefits by enhancing emotion regulation, reducing maladaptive cognitive patterns, and strengthening mindful parenting. This randomized controlled trial (RCT) examined the effectiveness of an eight-week Mindfulness-Based Cognitive Therapy (MBCT) program, enriched with mindful parenting practices, delivered to parents of children with ASD. The primary aim was to improve parental mental health, while secondary analyses explored potential indirect, parent-perceived changes in child behavior outcomes. **Methods**: Fifty-six parents of children with ASD were randomly assigned to an MBCT intervention group (*n* = 30) or a waitlist control group (*n* = 26). Parents completed assessments at baseline (T0), post-intervention (T1), and at one-month follow-up (T2), including the Depression Anxiety Stress Scales–21 (DASS-21), the Positive and Negative Affect Schedule (PANAS), and the Satisfaction With Life Scale (SWLS). They also rated the overall severity of their child’s behavior problems to explore indirect treatment effects. **Results**: All parents receiving MBCT (100%) completed the program successfully and reported high acceptability. At baseline, no significant differences were observed between groups. At T1, the MBCT group demonstrated significant reductions in depression, anxiety, and stress, alongside increases in positive affect and life satisfaction. These improvements were maintained or strengthened at T2. However, the control group showed no significant changes over time. Additionally, parents in the MBCT group reported indirect improvements in their children’s behavioral adjustment at T1 and T2. **Conclusions**: Findings demonstrate that MBCT constitutes an effective intervention for reducing parental psychopathology and indirectly supporting parent-perceived improvements in child behavior, emphasizing the importance of incorporating mindfulness and mindful parenting components into family-centered interventions for parents of children with ASD.

## 1. Introduction

### 1.1. Parental Mental Health of Children with Autism Spectrum Disorder

Autism Spectrum Disorder (ASD) is a multifactorial and lifelong neurodevelopmental disability that not only affects the individuals diagnosed but also imposes substantial and persistent mental health challenges on their families. The core features of ASD—difficulties in social communication and interaction, along with restricted and repetitive patterns of behavior—often lead to considerable limitations in functional independence and sustained caregiving demands across the lifespan [1,2]. As many individuals with ASD require ongoing specialized health, educational, and rehabilitation services, a substantial body of research has documented significant psychological consequences for caregivers, including diminished quality of life, emotional exhaustion, and increased social isolation [3,4,5].

Parenting, even under typical conditions, is an inherently challenging and emotionally complex process. Although most parents undertake this role with dedication and warmth, they often experience elevated parenting-related stress that is further intensified when either the parent or the child presents with symptoms of psychopathology, such as anxiety, depression, or behavioral dysregulation [6]. Developmental disability research consistently shows that approximately one-third of parents of children with ASD report notably elevated levels of stress, anxiety, and depressive symptoms compared to parents of neurotypical children [7,8,9]. In fact, Davis and Carter [10] found that 33% of mothers and 17% of fathers raising a child with ASD endorsed clinically significant depressive symptoms, while 6% in both groups reported clinically significant anxiety. Additionally, Carter et al. [11] found that maternal depressive symptoms among mothers of young children with ASD remained relatively stable over time, and that children’s behavior problems emerged as a key predictor of subsequent increases in parental psychopathology. In this framework, parental depression has been consistently associated with disruptions in parent–child interactions, including increased intrusiveness and reduced emotional sensitivity [12], which in turn are linked to adverse developmental outcomes in children across emotional social, and cognitive domains [13,14,15] as well as reduced learning capacity [16].

Raising a child with ASD is a chronic and enduring source of parental stress, often exceeding the burden reported by caregivers of children with other developmental disabilities such as Down syndrome, ADHD, and impairments in intellectual and adaptive functioning [17,18,19,20,21]. Consistent with these findings, Chen et al. [22], in a large cross-sectional study of 1450 parents, demonstrated that caregivers of children with ASD experienced significantly greater mental health difficulties than parents of children with intellectual or sensory disabilities. More recently, Papadopoulos [23] found that parents of children with ASD experienced higher emotional burden compared to parents of children with ADHD or global developmental delay. Notably, the strongest predictors of parental psychopathology were lower socioeconomic status and dysfunctional child behaviors rather than ASD severity. These results align with extensive evidence showing that child behavioral dysregulation is one of the most potent and consistent contributors to parental stress, symptoms of depression, and emotional exhaustion in families of children with ASD [2,4]. Behavioral difficulties—such as irritability, aggression, tantrums, self-injury, and non-compliance—place sustained demands on caregivers, often exceeding the challenges associated with core ASD symptoms alone [24,25]. In this regard, previous studies have demonstrated that these behavior problems not only predict higher levels of parental stress and depression but also exacerbate feelings of helplessness, reduce parenting efficacy, and strain family functioning [26,27]. Moreover, child behavior problems are among the strongest predictors of caregiver burnout and service utilization, reflecting their pervasive and cumulative impact on daily routines and emotional well-being [28].

Psychological distress has also been associated with increased marital dissatisfaction and a higher likelihood of marital dissolution [29,30]. Research consistently shows that the chronic psychological burden of raising a child with ASD can strain partner relationships, contributing to higher conflict, reduced emotional intimacy, and lower perceived relationship quality [31,32]. Couples raising children with ASD often report more disagreements about parenting responsibilities, financial strain, and reduced time for shared activities, all of which predict poorer marital functioning [33,34]. Marital distress, in turn, can exacerbate parenting stress and diminish the emotional resources available for effective caregiving. For single parents, the absence of co-parenting support may further intensify the stress associated with ASD-related caregiving demands, heightening vulnerability to psychological distress and burnout.

Parental psychological well-being represents a central component of the family’s developmental ecology [35]. Longitudinal research provides strong support for this transactional model, demonstrating that parental emotional functioning and child behavior dynamically influence each other over time [36,37]. A consistent bidirectional association has been documented between parenting stress and child behavior problems [38], forming a coercive and mutually reinforcing cycle of dysfunctional interaction. In this regard, higher levels of child behavior problems contribute to increased parental stress, which in turn predicts a subsequent exacerbation of behavioral difficulties. For example, Neece et al. [37] reported that parenting stress and child behavior problems significantly predicted one another through multiple developmental phases whereas elevated early parenting stress has been linked to poorer later child social skills [39,40].

Compared to parents of typically developing children, research suggests that parents of children with ASD exhibit distinct personality and socio-emotional profiles that may influence how they cope with the demands of caregiving. Li et al. [41] noted that these parents tend to show lower levels of novelty seeking and adaptability to change, as well as reduced social engagement, characteristics that may heighten stress vulnerability in unpredictable caregiving environments. Earlier work by Wolff et al. [42] also found that fathers of children with ASD displayed higher levels of schizoid traits relative to comparison groups, potentially reflecting broader familial patterns of social withdrawal or interpersonal detachment. More recent studies corroborate these observations, noting elevated levels of neuroticism, introversion, and reduced emotional stability among parents of children with ASD [43,44]. These personality characteristics have been associated with increased psychopathology, greater sensitivity to child behavior problems, and a tendency toward less flexible coping strategies. Conversely, protective traits such as emotional intelligence, resilience, conscientiousness, and extraversion have been linked to lower parenting stress and more adaptive responses to daily challenges [45,46]. Collectively, this literature suggests that parents’ dispositional factors significantly shape their psychological adjustment and coping skills when raising a child with ASD.

### 1.2. Mindfulness and Parental Mental Health

Mindfulness—classified within the third wave of Cognitive–Behavioral Therapies (CBT) [47,48,49]—constitutes a set of practices aimed at cultivating present-moment awareness of thoughts, emotions, and bodily sensations with an attitude of openness and nonjudgmental acceptance [50,51]. These characteristics render mindfulness a particularly practical and relevant avenue for caregivers who face persistent stressors, chronic emotional demands, and elevated caregiving burdens associated with raising a child with ASD. At the neurocognitive level, mindfulness practices have been linked to systematic changes in neural circuits involved in attentional control, emotional regulation, and self-referential processing, contributing to improvements in affect, well-being, and adaptive behavioral functioning [52,53].

A growing body of evidence demonstrates that mindfulness training can reduce parental stress and depressive symptoms, improve parents’ perceptions of their interactions with their children, and decrease both internalizing and externalizing child behaviors [38,54,55]. MBCT originally developed as an eight-week relapse-prevention program for individuals with recurrent depression [56,57], integrates mindfulness meditation with cognitive therapy strategies to help individuals identify maladaptive cognitive patterns, interrupt ruminative thought cycles, and cultivate greater emotional flexibility. Research findings suggest that MBCT may also positively influence parenting by reducing emotional reactivity, promoting empathic attunement, and increasing constructive engagement in parent–child interactions [58].

Mindful Parenting, introduced by Kabat-Zinn and Kabat-Zinn [59], extends mindfulness principles into the parenting context by emphasizing intentional, present-centered, and compassionate engagement with both the child and the parenting experience. This relational framework highlights attunement, acceptance, emotional awareness, and non-reactivity as core mechanisms that support healthier family relationships. Empirical studies show that Mindful Parenting programs strengthen parent–child relationships, reduce parental preoccupation and parenting stress, enhance children’s executive functioning, and promote more adaptive co-parenting interactions [60]. Importantly, Mindful Parenting has been associated not only with improvements in parental functioning but also in children’s attention and behavioral regulation, especially among those with externalizing or attentional difficulties [54,61,62]. Moreover, studies suggest that parents of children with ASD who endorse elevated levels of dispositional mindfulness report lower levels of stress, anxiety, and depression, as well as fewer behavioral problems and ASD-related symptoms in their children [63,64].

In summary, mindfulness-based interventions appear theoretically well-grounded and clinically appropriate for parents of children with ASD, as well as for parents experiencing clinically significant depressive symptoms or heightened vulnerability to psychological distress. By fostering acceptance, emotional regulation, and present-focused awareness, mindfulness practices may help parents interrupt maladaptive cycles of experiential avoidance and reactive parenting while strengthening their capacity for attuned, sensitive, and compassionate caregiving. Nevertheless, further experimental research—particularly from diverse cultural contexts—is needed to clarify the direct and long-term effects of mindfulness interventions on parental well-being, parenting processes, and child developmental outcomes.

### 1.3. Current Study

According to the above evidence, the aim of the present RCT was to evaluate the preliminary efficacy of MBCT for parents of children aged 4–17 years with ASD who reported at least mild symptoms of depression, anxiety, or stress. The intervention preserved the core structure of standard MBCT while integrating mindful parenting elements designed to enhance mindful awareness specifically with emphasis on reducing emotional reactivity, fostering acceptance and empathy, and strengthening parental engagement. Importantly, the program did not include explicit behavioral parenting strategies. In contrast, it focused on applying mindfulness principles to daily parenting challenges which are often characterized by heightened emotional fatigue and may contribute to the intergenerational transmission of depressive vulnerability.

The present study examined the effects of MBCT on parental mental health and explored potential indirect parent-perceived changes in child behavioral outcomes in an outpatient mental health care setting. We hypothesized that: (a) parents in the MBCT group would exhibit significant reductions in depressive, anxiety, and stress symptoms, as well as significant increases in positive affect and life satisfaction, both immediately post-intervention and at one-month follow-up, compared to parents in the waitlist control group; (b) parents in the MBCT group would demonstrate high intervention acceptability; and (c) children of parents in the MBCT group would show greater improvements in parent reported severity of child’s behavior problems relative to those in the waitlist control group.

## 2. Materials and Methods

### 2.1. Study Design and Setting

The study used a two-arm RCT comparing an intervention group receiving MBCT with a waitlist-control group. Assessments were conducted at baseline (T0), immediately post-intervention (T1), and at one-month follow-up (T2) to evaluate both the immediate effects and short-term maintenance of treatment outcomes. Participants were randomly allocated to conditions using simple randomization procedures to minimize selection bias and ensure comparable groups. The study design followed key CONSORT recommendations—such as random allocation and clearly defined assessment timepoints; thereby, enhancing the methodological clarity and interpretability of the findings.

The study was conducted between February 2024 and April 2025 in an outpatient mental health clinic in Athens, Greece. This setting offered a structured, supportive, and therapeutically appropriate environment for family-focused interventions. All sessions and assessments were carried out in designated therapeutic rooms to ensure privacy, consistency, and adherence to professional standards.

This study was part of a postdoctoral research project and received ethical approval from the Ethics and Research Committee of the University of West Attica (Approval No. 8119/12-02-2024). Additional authorization for implementation of the intervention was granted by the administrative board of the collaborating mental health clinic (Approval No. 661/11-12-2023). All study procedures complied with the ethical principles outlined in the Declaration of Helsinki for research involving human participants.

### 2.2. Participants and Sample Size

**Figure 1:**
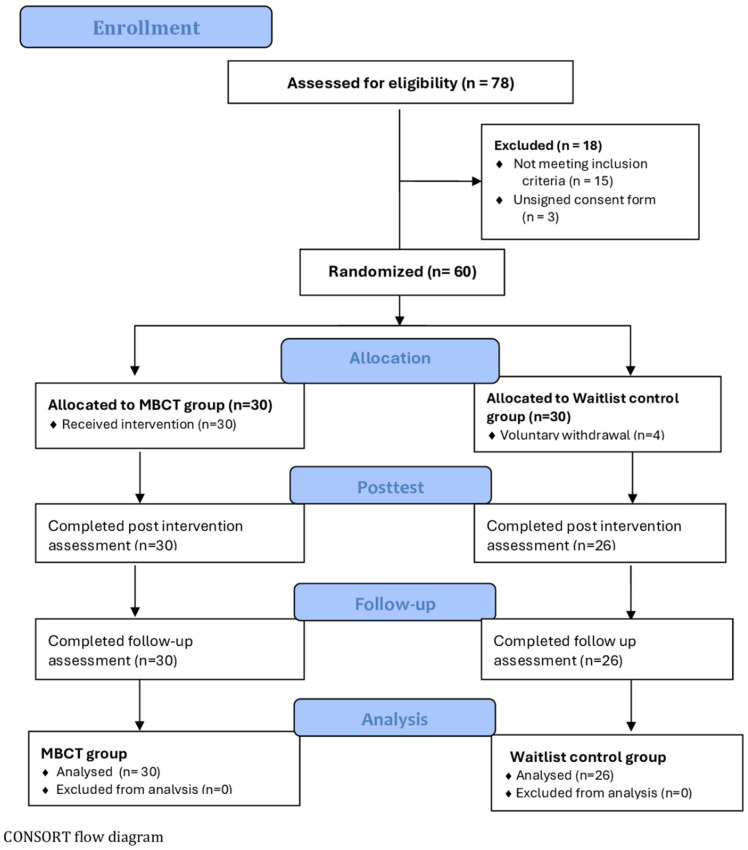
Flow chart of the study.

A total of 78 Greek parents were initially screened for eligibility through multiple recruitment sources, including primary care settings, outpatient mental health clinics, parent associations in local primary schools, and community outreach initiatives (e.g., social media announcements, flyers, public advertisements). Eighteen individuals were excluded during screening due to insufficient DASS-21 symptom severity, concurrent psychotherapy participation, or refusal to provide informed consent. The remaining 60 eligible parents were enrolled and randomly assigned to either the MBCT intervention group (*n* = 30) or the waitlist-control group (*n* = 30). Figure 1 summarizes participant flow through the study following CONSORT guidelines.

An a priori power analysis was conducted using G*Power 3.1.9.4 [65] to determine the required sample size. The analysis was based on a repeated-measures ANOVA examining the group × time interaction (two groups; three assessment points: T0, T1, T2). Assuming a medium effect size (f = 0.25), α = 0.05, and desired power of 0.96, a minimum of 46 participants was required. Due to potential attrition, we sought to recruit at least 20% more participants, targeting an overall sample of 56 parents.

### 2.3. Inclusion and Exclusion Criteria

To be included in the study, individuals were required to meet the following criteria: (a) be a parent and primary caregiver aged ≥18 years and have at least one child diagnosed with ASD, aged 4 to 17 years, living in the same household; and (b) have sufficient fluency in Greek to understand instructions and complete all study procedures. To ensure parents most likely to benefit from the intervention, eligible participants were also required to present at least mild symptoms of depression, anxiety, or stress, based on the established DASS-21 cut-offs [66]: Depression ≥ 10, Anxiety ≥ 8, or Stress ≥ 15.

Exclusion criteria included participation in individual or group Cognitive Behavioral Therapy (CBT) within the previous 12 months, to maintain sample homogeneity and minimize confounding treatment effects. Participants were also required to attend at least 80% of MBCT sessions (i.e., ≥6 out of 8) to be included in post-intervention (T1) and follow-up (T2) assessments. However, the exclusion criterion based on attendance was not applied as no participants in the intervention group dropped out or failed to meet this attendance requirement.

### 2.4. Measures

#### 2.4.1. Psychosocial and Demographic Questionnaire

Participants completed a sociodemographic questionnaire developed by the researchers to provide a comprehensive assessment of individual and family characteristics. The first section collected parent-related information, including gender, age, marital and employment status, as well as educational attainment. The second section concerned the child, gathering data such as gender, age, and time since diagnosis. Parents also rated their child’s overall behavior using a four-point ordinal scale (0 = “no behavioral problems” to 3 = “severe behavioral problems”), a brief approach previously used in ASD research [28]. This rating reflected parents’ impressions of the child’s everyday behavioral functioning, considering common domains such as aggression, anxiety-related behaviors, regulatory difficulties, and non-compliance.

#### 2.4.2. Parental Mental Health and Wellbeing

##### Depression Anxiety and Stress Scale (DASS-21) [66]

The DASS-21 is a self-report instrument comprising 21 items divided into three subscales: Depression, Anxiety, and Stress. Items are rated on a four-point Likert scale (0 = “Did not apply to me at all” to 3 = “Applied to me very much or most of the time”). Subscale scores are calculated by summing the relevant items and multiplying by two to ensure comparability with the full DASS-42. The DASS-21 has been widely used with parents of children with developmental disabilities [67,68] and has been validated in the Greek population [69]. In the present study, internal consistency coefficients (Cronbach’s α) were α = 0.75 for Depression, α = 0.64 for Anxiety, and α = 0.82 for Stress, indicating acceptable to good reliability (see Table 1).

##### Satisfaction with Life Scale (SWLS) [70]

The SWLS assesses overall life satisfaction through five items rated on a seven-point Likert scale (1 = strongly disagree to 7 = strongly agree). Total scores range from 5 to 35, with higher scores indicating greater life satisfaction. Suggested interpretive categories include: 5–9 (extremely dissatisfied), 10–14 (dissatisfied), 15–19 (below average satisfaction), 20–24 (moderately satisfied), 25–29 (quite satisfied), and 30–35 (very satisfied). The Greek adaptation demonstrates satisfactory psychometric properties [71]. As shown in Table 1, Cronbach’s α in the present study was 0.82, indicating good internal consistency.

##### Positive and Negative Affect Scale (PANAS) [72]

The PANAS consists of 20 items that measure the frequency of positive affect (10 items) and negative affect (10 items) on a five-point Likert scale (1 = very slightly or not at all to 5 = extremely). The instrument has been widely used in research involving parents of children with disabilities [73]. The scale has been translated and adapted into Greek with adequate psychometric properties [74]. Table 1 presents the internal consistency coefficients for the current study, which were α = 0.63 for Positive Affect and α = 0.77 for Negative Affect.

**Table 1:** Summary of instruments used in the study.

Instruments	Items/Subscales	Scoring and Interpretation	Cronbach’s α (Present Study)
DASS-21 [66] (Depression, Anxiety, Stress)	21 3 subscales (7 items each)	4-point Likert scale (0–3); subscale scores multiplied by 2; higher scores indicate greater symptom severity.	Depression = 0.75, Anxiety = 0.64, Stress = 0.82
PANAS [70] (Positive–Negative Affect)	20; 2 subscales (10 items each)	5-point Likert scale (1–5); higher PA = greater positive affect, higher NA = greater distress.	Pos. = 0.63 Neg. = 0.77
SWLS [72] (Satisfaction with Life)	5	7-point Likert scale (1–7); higher total scores reflect greater life satisfaction.	Life satisfaction = 0.82

#### 2.4.3. Intervention Acceptability

Acceptance of the intervention was assessed using a brief study-specific questionnaire developed by the researchers based on relevant literature on mindfulness interventions. Parents evaluated important program components (e.g., session structure, content relevance, home practice activities, facilitator support) on a five-point Likert scale (1 = strongly disagree, 5 = strongly agree). Participants were asked to provide qualitative feedback on their overall experience by responding to two more open-ended questions about the program’s most and least beneficial elements.

### 2.5. Procedure

Parents who expressed interest in the study were contacted by email or telephone and completed an eligibility screening, which included confirmation of their child’s ASD diagnosis. The study’s objectives, methods, and procedure were thoroughly described to parents who met the inclusion criteria at an online or in-person briefing session. Informed consent was obtained prior to participation. The study followed CONSORT recommendations for RCTs. 

After completing baseline assessments, participants were randomly allocated to the MBCT intervention group (*n* = 30) or the waitlist-control group (*n* = 30) using a com-puter-generated randomization sequence to minimize selection bias. Outcome measures were collected via self-administered questionnaires completed independently by par-ticipants, without direct interaction with the research team, reducing the likelihood of assessment bias related to awareness of group assignment. Data entry and dataset preparation were performed by a researcher blinded to group assignment to minimize analytic bias. 

Participants allocated to the MBCT group proceeded to the intervention phase (described in Section 2.6). To maintain consistency and enhance group cohesion, the 30 participants allocated to the MBCT condition were organized into two fixed subgroups of 15 individuals each, which remained stable throughout the eight-week program. Participants in the waitlist-control group continued with treatment as usual.

Post-intervention assessments (T1) were conducted within one week following the final session, and follow-up assessments (T2) were administered four weeks later to examine short-term maintenance of treatment effects. All assessments were completed individually. All data were anonymized using unique identification codes and stored on encrypted, password-protected systems in accordance with GDPR requirements.

In the intervention group, all 30 parents initiated and completed the eight-week MBCT program without attrition and all participants satisfied the predefined ≥80% attendance criterion. In the waitlist control group, 26 of 30 parents completed both T1 and T2 assessments; four participants withdrew immediately after randomization and completed only the baseline assessment. Thus, the final analytic sample comprised 56 parents (MBCT: *n* = 30; control: *n* = 26). All exclusions were due solely to missing follow-up data rather than intervention-related reasons.

### 2.6. Intervention

The intervention followed the standard eight-week MBCT protocol [56,57], delivered in weekly two-hour group sessions. MBCT integrates cognitive therapy principles with mindfulness meditation practices to cultivate present-moment awareness, reduce automatic negative thinking, enhance emotional regulation, and strengthen self-compassion, thereby enabling participants to experience greater psychological wellbeing.

To ensure relevance for parents of children with ASD, the program incorporated established mindful parenting components [6,59,75], without altering the core MBCT curriculum. These additions emphasized applying mindfulness to real-time parenting challenges, including recognizing emotionally driven parenting reactions, increasing awareness of the parent–child relational dynamic, cultivating acceptance, and promoting nonreactive responding in high-stress caregiving situations.

Each session included guided mindfulness practices (e.g., body scan, sitting meditation, mindful movement), compassion-focused exercises toward self and child, cognitive-behavioral elements (identifying automatic thoughts, cognitive reactivity, and decentering), experiential activities, and structured group inquiry linking personal practice experiences to MBCT principles. Table 2 presents the program’s structure and content. Participants were assigned 30–40 min of daily home practice supported by Greek-translated audio recordings and written materials. To encourage participation and skill consolidation, home practice was reviewed weekly.

Sessions were facilitated by the first author, an academic specializing in developmental psychology and developmental psychopathology with accredited training in mindfulness-based interventions and clinical psychotherapeutic practice. Attendance was recorded, with an expectation of ≥80% participation to ensure adequate exposure. Participants in the waitlist-control group continued treatment as usual and were offered the MBCT program after completing T2. Attendance was recorded to ensure adequate exposure and after completing T2, the MBCT program was offered to participants in the waitlist-control group who continued to receive standard treatment.

**Table 2:** Overview of the MBCT Intervention: Session-by-Session Protocol.

Week/Session	Core Theme	Primary Mindfulness Practices	MBCT/Mindful Parenting Components
**Week 1**	Awareness and Autopilot	Mindful eating (raisins task); Introduction to body scan	Recognizing automatic parenting reactions; introducing the program structure and group guidelines
**Week 2**	Facing Obstacles/A Different Way of Knowing	Body scan; Short mindful breathing	Distinguishing “doing” vs. “being” mode; observing difficult parenting thoughts/emotions as mental events
**Week 3**	Focusing the Scattered Mind	Extended mindful breathing; Mindful movement; 3-min breathing space	Applying attentional focus before/after challenging parent–child interactions
**Week 4**	Responding to Experience/Recognizing Aversion	Sitting meditation (breath, body, sounds, thoughts)	Noticing pleasant and unpleasant events in parenting; cultivating nonjudgmental awareness toward the child
**Week 5**	Allowing and Letting Be	Acceptance-focused meditation (observing difficult emotions)	Allowing parental emotions (guilt, frustration, sadness) to arise without avoidance or suppression
**Week 6**	Thoughts Are Not Facts	Sitting meditation emphasizing thought awareness	Decentering from self-critical thoughts (e.g., “I am not a good parent”); reframing automatic interpretations
**Week 7**	Self-Care and Relapse Prevention	Extended sitting meditation	Identifying personal stress triggers; developing a self-care and mindful parenting maintenance plan
**Week 8**	Maintaining and Extending New Learning	Review of core practices; Choice of preferred meditation	Creating individualized mindful parenting plans; integrating mindfulness into daily routines

### 2.7. Statistical Analysis

All statistical analyses were conducted using IBM SPSS Statistics (Version 25). Assumptions of normality were evaluated through skewness and kurtosis values, all of which fell within the acceptable ±2 range, supporting the use of parametric procedures [76,77]. This approach aligns with other RCTs in the mindfulness literature [78].

Descriptive statistics were computed for all sociodemographic and baseline clinical variables, and internal consistency (Cronbach’s α) was examined for each psychometric instrument. Independent-samples *t*-tests for continuous variables and chi-square tests for categorical variables were used to evaluate baseline equivalence between the MBCT and waitlist-control groups.

Intervention effects were examined using repeated-measures ANOVAs with time (T0, T1, T2) as the within-subjects factor and group (MBCT vs. waitlist-control) as the between-subjects factor. The primary focus of inference was the group × time interaction. Partial eta squared (*η*^2^*p*) was reported as the effect size for ANOVA models. When significant interactions were detected, post hoc paired-samples *t*-tests evaluated within-group changes across time, and independent-samples *t*-tests compared groups at each assessment point. For within-group comparisons, effect sizes were calculated using Cohen’s *d* for paired samples (*d_z_*), whereas standard Cohen’s *d* was used for between-group comparisons.

Changes in parent-reported child behavior problem severity over time and between groups were analyzed using chi-square tests, and effect sizes were measured using Cramer’s V. Statistical significance was set at α = 0.05 (two-tailed).

## 3. Results

### 3.1. Sociodemographic and Clinical Characteristic

Table 3 presents the baseline sociodemographic characteristics of participating parents and their children. There were no statistically significant differences between the MBCT and waitlist-control groups on any demographic category (all *p* > 0.05), indicating appropriate baseline equivalence. Most parents (66.1%) were mothers and were married. Nearly half (42.9%) had received technical or technological education. The mean age of the parents was 40.67 years (SD = 6.45), and the two groups did not differ in age, gender distribution, or employment status. In terms of child characteristics, most children (80.4%) were males, with a mean age of 10.54 years and an average time since ASD diagnosis of 5.84 years. Similar to parental factors, no statistically significant between-group variations were found in child sociodemographic variables at baseline.

Baseline clinical outcomes are shown in Table 4. All mental health indicators showed no significant between-group differences (all *p* > 0.05). Parents were classified within mild to moderate symptom severity range on the DASS-21 (M_depression_ = 17.32; M_anxiety_ = 13.00, and M_stress_ = 26.14). Life satisfaction scores were low (M = 15.71) and fell into the category of “slightly dissatisfied”. Similarly, PANAS scores across groups showed a clinically significant trend of increased negative affect (M = 22.80) and decreased positive affect (M = 27.79). At baseline (T0) (*p* = 0.923), parent-reported severity of child behavioral problems did not differ significantly between groups, suggesting that both groups had comparable clinical profiles at the start of the study. The homogeneity of the overall sample strengthens the methodological validity of subsequent between-group comparisons.

**Table 3:** Social and Demographic Characteristics of Participants.

Variables	Total Participants (*n* = 56)	MBCT Group (*n* = 30)	Waitlist Control Group (*n* = 26)	*χ*^2^/*t*	*p*
**Parental characteristics**					
Gender, *n* (%)					
Male	19	9	10		
Female	37	21	16	0.445	0.505
Age in years (M/SD)	40.67	40.52	40.85	−0.194	0.846
Marital status, *n* (%)					
Married	42	22	20 (76.9%)		
Divorced	9	4	5		
Single parent	5	4	1	1.729	0.421
Education level, *n* (%)					
Secondary	10	3	7		
Technical/Technological	24	15	9		
University	22	12	10	3.011	0.222
Employment status, *n* (%)					
Public employee	16	7	9		
Private employee	27	15	12		
Self-employed	13	8	5	0.995	0.608
**Child characteristics**					
Gender, *n* (%)					
Boy	45	24	21		
Girl	11	6	5	0.005	0.942
Age in years (M/SD)	10.54	10.51	10.59	0.072	0.942
Time since diagnosis (years) (M/SD)	5.84	5.78	5.9	−0.129	0.897
Severity of child’s behavior problem, *n* (%)					
Mild	9 (16.1%)	5 (16.7%)	4 (15.4%)		
Moderate	20 (35.7%)	10 (33.3%)	10 (38.5%)		
Severe	27 (48.2%)	15 (50%)	12 (46.2%)	0.160	0.923

**Table 4:** Baseline Clinical Characteristics of Participants.

Indicators	Total Participants (*n* = 56)	MBCT Group (*n* = 30)	Control Group (*n* = 26)	*t*	*p*
	M/SD	M/SD	M/SD		
**DASS-21**					
Depression	17.32 ± 4.29	17.40 ± 4.85	17.23 ± 3.63	0.150	0.882
Anxiety	13.00 ± 2.70	12.93 ± 3.27	13.08 ± 1.90	−0.210	0.832
Stress	26.14 ± 4.73	26.13 ± 5.01	26.15 ± 4.49	−0.021	0.987
**PANAS**					
Positive Affect	27.79 ± 2.10	27.70 ± 2.45	27.88 ± 1.65	−0.334	0.746
Negative Affect	22.80 ± 2.70	23.03 ± 3.35	22.54 ± 1.70	0.710	0.485
**SWLS**					
Life satisfaction	15.71 ± 2.81	15.60 ± 3.16	15.85 ± 2.41	−0.340	0.739

### 3.2. Effect of the MBCT Program on Parental Mental Health Outcomes

#### 3.2.1. Depression

**Figure 2:**
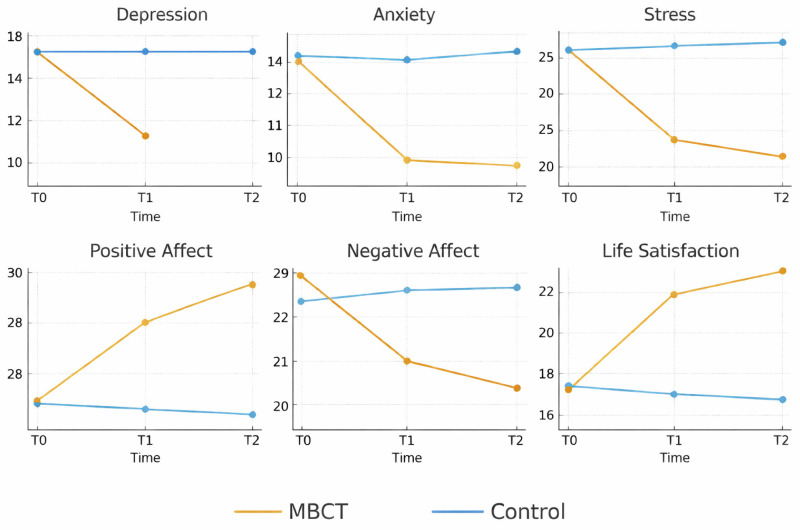
Changes in Parental Mental Health Outcomes Across Time by Group.

Repeated-measures ANOVA revealed significant main effects of group (*F* = 22.950, *p* < 0.001, *η*^2^*p* = 0.298) and time (*F* = 50.344, *p* < 0.001, *η*^2^*p* = 0.482) on depressive symptoms. Importantly, a Group × Time interaction emerged (*F* = 64.126, *p* < 0.001, *η*^2^*p* = 0.543). As presented in Table 5, within-group comparisons indicated substantial reductions in depression in the MBCT group from baseline to post-intervention and from baseline to follow-up suggesting continued symptomatic relief over time. In contrast, the waitlist control group showed no significant changes in depression levels across assessments. Figure 2 illustrates that, the MBCT group reported significant lower depressive symptoms than the control group at post-intervention, with this difference being even more pronounced at follow-up.

#### 3.2.2. Anxiety

Repeated-measures ANOVA revealed significant main effects of group (*F* = 21.730, *p* < 0.001, *η*^2^*p* = 0.287) and time (*F* = 18.462, *p* < 0.001, *η*^2^*p* = 0.255), as well as a significant Group × Time interaction (*F* = 29.298, *p* < 0.001, *η*^2^*p* = 0.352). As shown in Table 5, participants in the MBCT group exhibited marked reductions in anxiety from baseline to post-intervention, followed by additional improvements at follow-up. No additional change was observed between post-intervention and follow-up, indicating stability of treatment gains over time. Conversely, no comparable changes were observed in the control group. Be-tween-group comparisons further indicated that the MBCT group reported significantly lower anxiety levels than the control group at post-intervention, with anxiety levels continuing to decline at follow-up (see Figure 2). 

#### 3.2.3. Stress

A repeated-measures ANOVA revealed significant main effects of group (*F* = 25.530, *p* < 0.001, *η*^2^*p* = 0.321) and time (*F* = 143.828, *p* < 0.001, *η*^2^*p* = 0.727), as well as a significant Group × Time interaction (*F* = 188.515, *p* < 0.001, *η*^2^*p* = 0.777). As shown in Table 5, within-group analyses indicated substantial reductions in stress in the MBCT group from baseline to post-intervention. A smaller but statistically significant improvement was also observed between post-intervention and follow-up, suggesting continued symptom improvement over time. In comparison, the waitlist control group exhibited no significant changes in stress across any of the three assessment points. Between-group comparisons further supported the effectiveness of the intervention, with lower stress levels observed in the MBCT group compared to the control group at post-intervention and follow-up (see Figure 2). 

#### 3.2.4. Life Satisfaction

Repeated-measures ANOVA revealed significant main effects of group and time, as well as a robust Group × Time interaction (Fs = 62.429 and 87.903 respectively, ps < 0.001; interaction: *F* = 132.156, *p* < 0.001). According to Table 5, within-group analyses indicated substantial increases in life satisfaction in the MBCT group from baseline to post-intervention, followed by additional increases at follow-up. However, no significant changes emerged at any time point in the waitlist control group. Figure 2 shows that parents in the MBCT group reported higher levels of life satisfaction than those in the control group at post-intervention, with this difference remained evident at follow-up. 

#### 3.2.5. Positive and Negative Affect

For positive affect, analyses revealed a significant main effect of group (*F* = 9.725, *p* = 0.003, *η*^2^*p* = 0.153) and time (*F* = 23.866, *p* < 0.001, *η*^2^*p* = 0.306), as well as a significant Group × Time interaction (*F* = 32.066, *p* < 0.001, *η*^2^*p* = 0.373). As reported in Table 5, within-group analyses indicated that parents in the MBCT group showed marked increases in positive affect from baseline to post-intervention, with further gains maintained at follow-up. No significant changes over time were observed in the control group. Between-group comparisons further demonstrated that the MBCT group reported higher levels of positive affect than the control group at both post-intervention and follow-up assessments (see Figure 2). 

As illustrated in Table 5, a significant main effect of group (*F* = 7.292, *p* = 0.009, *η*^2^*p* = 0.119) and time (*F* = 14.189, *p* < 0.001, *η*^2^*p* = 0.208), as well as a significant Group × Time interaction (*F* = 25.267, *p* < 0.001, *η*^2^*p* = 0.319), was revealed. Within-group analyses indicated that the MBCT group showed consistent reductions in negative affect from baseline to post-intervention and this effect remained evident at follow-up. In the control group, however, no significant changes were observed across the three assessment points. According to Figure 2, between-group comparisons (further demonstrated that the MBCT group reported significantly lower negative affect than the control group at post-intervention with the advantage persisting and strengthening at follow-up. 

**Table 5:** Comparisons of Parental Mental Health Outcomes by Group and Time.

Indicator	Group	M ± SD (T0)	M ± SD (T1)	M ± SD (T2)	T0 vs. T1	T0 vs. T2	T1 vs. T2	Group × Time Interaction (*F*, *p*)
**Depression**	MBCT	17.40 ± 4.85	11.77 ± 2.67	11.00 ± 2.39	*t*(29) = 10.37, *p* < 0.001, *d* = 1.89	*t*(29) = 8.52, *p* < 0.001, *d* = 1.56	*t*(29) = 2.37, *p* = 0.020, *d* = 0.43	*F* = 64.126, *p* < 0.001, *η*^2^*p* = 0.543
	Control	17.23 ± 3.63	17.46 ± 3.28	17.69 ± 3.47	*t*(25) = −1.14, *p* = 0.265, *d* = −0.22	*t*(25) = −1.54, *p* = 0.136, *d* = −0.30	*t*(25) = −0.90, *p* = 0.376, *d* = −0.18	
**Anxiety**	MBCT	12.73 ± 3.50	9.40 ± 2.24	9.30 ± 2.09	*t*(29) = 7.53, *p* < 0.001, *d* = 1.37	*t*(29) = 6.72, *p* < 0.001, *d* = 1.23	*t*(29) = 0.40. *p* = 0.687, *d* = 0.07	*F* = 29.298, *p* < 0.001, *η*^2^*p* = 0.352
	Control	12.92 ± 2.05	13.15 ± 2.05	13.46 ± 2.16	*t*(25) = 1.14, *p* = 0.265 *d* = −0.22	*t*(25) = −1.54, *p* = 0.136, *d* = −0.30	*t*(25) = −0.90, *p* = 0.376, *d* = −0.18	
**Stress**	MBCT	26.13 ± 5.01	18.93 ± 4.48	17.30 ± 4.09	*t*(29) = 16.96, *p* < 0.001, *d* = 3.10	*t*(29) = 17.56, *p* < 0.001, *d* = 3.21	*t*(29) = 4.18, *p* < 0.001, *d* = 0.76	*F* = 188.515, *p* < 0.001, *η*^2^*p* = 0.777
	Control	26.15 ± 4.09	26.46 ± 4.09	26.85 ± 3.76	*t*(25) = −0.39, *p* = 0.800, *d* = −0.05	*t*(25) = −1.17, *p* = 0.025, *d* = −0.23	*t*(25) = −0.65, *p* = 0.500, *d* = −0.13	
**Positive** **Affect**	MBCT	27.70 ± 2.45	29.97 ± 2.34	30.43 ± 2.39	*t*(29) = −6.38, *p* < 0.001, *d* = −1.16	*t*(29) = −6.46, *p* < 0.001, *d* = −1.18	*t*(29) = −2.45, *p* = 0.020, *d* = −0.45	*F* = 32.066, *p* < 0.001, *η*^2^*p* = 0.373
	Control	26.69 ± 3.96	27.88 ± 1.66	27.65 ± 1.65	*t*(25) = −1.28, *p* = 0.211, *d* = −0.25	*t*(25) = −1.01, *p* = 0.323, *d* = −0.20	*t*(25) = 1.19, *p* = 0.247, *d* = 0.23	
**Negative Affect**	MBCT	23.03 ± 3.35	20.83 ± 1.72	20.23 ± 1.92	*t*(29) = 4.41, *p* < 0.001, *d* = 0.80	*t*(29) = 5.34, *p* < 0.001, *d* = 0.98	*t*(29) = 3.67, *p* = 0.001, *d* = 0.67	*F* = 25.267, *p* < 0.001, *η*^2^*p* = 0.319
	Control	22.54 ± 1.70	22.81 ± 1.90	22.96 ± 1.97	*t*(25) = 0.97, *p* = 0.341, *d* = −0.19	*t*(25) = −1.53, *p* = 0.139, *d* = −0.30	*t*(25) = −1.69, *p* = 0.103, *d* = −0.33	
**Life** **Satisfaction**	MBCT	15.60 ± 3.16	21.77 ± 2.97	23.07 ± 2.49	*t*(29) = −12.50, *p* < 0.001, *d* = −2.29	*t*(29) = −14.80, *p* < 0.001, *d* = −2.71	*t*(29) = −4.71, *p* < 0.001, *d* = −0.86	*F* = 132.156, *p* < 0.001, *η*^2^*p* = 0.710
	Control	15.31 ± 1.69	15.12 ± 1.53	15.04 ± 1.93	*t*(25) = 0.48, *p* = 0.638, *d* = 0.09	*t*(25) = 1.32, *p* = 0.199, *d* = 0.26	*t*(25) = 0.20, *p* = 0.844, *d* = 0.04	

### 3.3. Intervention Acceptability

Parents receiving the MBCT intervention demonstrated very high levels of acceptability, with 96.4% reporting overall strong satisfaction with the program (M = 4.82, *SD* = 0.39). Regarding the structure and content of the intervention, 95.4% of participants indicated high satisfaction, reflecting strong approval of the program’s organization, session flow, and relevance to their personal and family needs. Similarly, 97.4% rated their overall experience and the suitability of the program as highly positive, highlighting the supportive group atmosphere, the facilitator’s guidance, and the practical usefulness of the skills taught. Open-ended responses also revealed that parents found the mindfulness practices highly appropriate for daily parenting and intended to continue using them beyond the program. The main obstacles identified were childcare needs and travel distance, while some parents suggested that future interventions could benefit from smaller group sizes to enhance interaction and personal sharing.

### 3.4. Child Behavioral Outcomes

**Figure 3:**
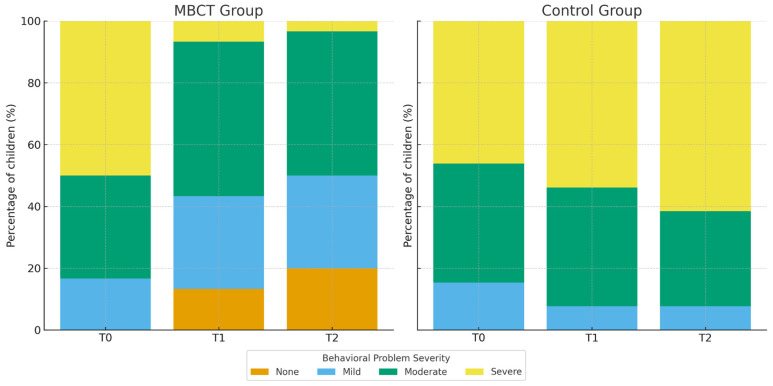
Comparison of Children’s Behavioral Problems Across Time by Group.

Because no significant between-group differences were observed in parent-reported child behavioral problems at baseline (T0) (*p* = 0.923; see Section 3.1), subsequent analyses focused on changes in parent-perceived child behavior at post-intervention (T1) and follow-up (T2). At T1, a statistically significant between groups difference was observed (*χ*^2^(3) = 18.262, *p* < 0.001), corresponding to a large effect size (Cramer’s *V* = 0.57). In the MBCT group, only 6.7% of children remained in the severe range while 13.3% were rated as having no behavioral problems. In contrast, in the waitlist-control group, 53.8% of children continued to exhibit severe behavioral difficulties and no child was rated as problem-free. This pattern was further strengthened at T2, where another significant group difference was revealed (*χ*^2^(3) = 25.169, *p* < 0.001; Cramer’s *V* = 0.67). As shown in Figure 3, the proportion of children rated as having no behavioral difficulties increased to 20% in the MBCT group, while only 3.3% remained in the severe category. Conversely, 61.5% of children in the control group continued to present with severe problems, and none were rated as having no difficulties

## 4. Discussion

### 4.1. Preliminary Findings

At baseline, the two groups were comparable in terms of all sociodemographic and clinical factors, confirming the adequacy of randomization. More specifically, parents had significantly higher levels of depression, anxiety, and stress, accompanied by reduced life satisfaction. These scores fell within clinically relevant ranges across important mental health indices and were significantly higher than normal population norms [79]. Such findings have been consistently reported in previous studies which demonstrate the elevated psychological burden experienced by parents of children with ASD and underline the clinical relevance of evaluating interventions specifically targeting this population [73,80,81]. Moreover, this high level of initial distress provided substantial scope for therapeutic improvement.

### 4.2. Effectiveness of MBCT on Parental Mental Health

The current study’s results suggest that the MBCT program was highly effective in reducing depressive symptoms, with parents in the MBCT group exhibiting significant gains both at immediately post-intervention and at follow-up. These findings align with a large body of research showing that MBCT reliable improves depressive symptoms and prevents relapse [56,57,82,83] while also enhancing psychological flexibility and resilience [84,85]. Depression is typically characterized by rigid negative schemas, ruminative thinking, and heightened attentional focus on distressing emotional states [86,87]. MBCT can reduce depressive symptomatology by interrupting the repetitive negative cognitive cycles that sustain emotional distress and depressive episodes; thereby fostering metacognitive awareness, cognitive flexibility, acceptance, and self-compassion. Accordingly, this cognitive shift enables individuals to cope with difficult situations with less automatic reactivity and greater psychological flexibility, functioning as a broad regulatory mechanism that yields downstream benefits across anxiety- and stress-related processes.

Within the context of parenting a child with ASD—an environment marked by chronic stressors and heightened emotional demands—these internal regulatory gains are likely further reinforced by the mindful parenting components embedded in the MBCT protocol. These practices foster present-moment awareness, emotional attunement, and compassion in parent–child interactions [60,75,88,89], which, in turn, help disrupt entrenched reactive patterns and reduce the likelihood that everyday challenges will elicit negative interpretations or self-critical rumination. Through this dual pathway—targeting both internal emotion-regulation processes and the interpersonal quality of parent–child exchanges—the combined MBCT and mindful parenting approach fosters a more regulated, attuned, and compassionate caregiving stance.

In terms of parental stress, the MBCT group showed both immediate post-intervention and long-term gains, with stress levels decreasing significantly from the severe range at baseline to the mild range at T1 and falling within the normative range by T2. These results are consistent with previous studies revealing that mindfulness-based interventions reliably reduce stress and enhance emotional functioning among caregivers of children with ASD [90,91,92,93]. Mindfulness may serve as a protective factor particularly for parents who must balance demanding caregiving responsibilities alongside professional pressures, financial strain, and persistent work–family conflict [94]. Parents receiving MBCT learn to interrupt automatic stress responses and shift attention toward more adaptive interpretations through practices that cultivate a non-judgmental present-moment stance, mindful disengagement from “autopilot”, and greater sensitivity to internal cues [95]. This in turn expands their capacity to cope with daily challenges in more flexible and adaptive ways. Indeed, such cognitive–emotional shifts reduce reliance on fight-or-flight patterns—commonly associated with anxiety and heightened reactivity [96]—and foster more deliberate, emotionally regulated parenting behaviors.

Significant improvements in perceived positive affect and life satisfaction were observed among participants in the MBCT group, who shifted from “somewhat dissatisfied” at baseline to “somewhat satisfied” at post-intervention and follow-up. Such findings align with evidence indicating that mindfulness practice reduces self-criticism, enhances positive emotion, and supports broader psychological flourishing [97,98,99]. According to the Mindfulness-to-Meaning Theory [95], mindfulness contributes to psychological well-being through conscious emotion regulation and disengagement from habitual negative attentional patterns, facilitating metacognitive awareness. Mindfulness practices that cultivate decentering and broaden attentional scope may initiate an upward spiral of adaptive cognitive–affective processing that progressively transforms how individuals respond to stress. This shift enables individuals to engage in more constructive cognitive reappraisal and to flexibly reinterpret both internal and external experiences, thereby enhancing their capacity to cope with adversity with greater clarity, emotional calmness, and a strengthened sense of purpose. More specifically, these cognitive processes are linked to enhance well-being primarily through their influence on affective functioning, including increased positive affect and reduced negative affect [100].

For families raising a child with ASD, this process may represent a central pathway linking mindfulness practice to improved life satisfaction [101] as parents become more capable of re-experiencing positive emotions and evaluating daily life more favorably. In turn this may foster a more compassionate and meaningful stance toward their caregiving role while reducing automatic negative appraisals. such as guilt or self-blame, related to the child’s disability and challenging behaviors. Within this framework, the Mindfulness-to-Meaning Theory helps conceptualize how mindfulness-based interventions may promote mindful parenting by interrupting automatic stress-driven reactions and fostering greater psychological flexibility [94]. Rather than reacting reflexively, parents may learn to pause, attend to present-moment experience, and broaden awareness to include alternative perspectives and coping options that may previously have been overlooked (e.g., identifying flexible strategies to manage work–family demands).

Several of the observed intervention effects were relatively large compared to those typically reported in psychosocial interventions, and this finding warrants careful consideration. One contributing factor may be the elevated levels of psychological distress observed at baseline, which likely provided greater scope for therapeutic improvement following intervention. Indeed, the present sample appeared to report increased psychological burden, reflecting the heightened vulnerability and unmet mental health needs of parents raising a child with ASD in Greece, a population that remains comparatively under-researched, and highlighting the chronic emotional demands associated with caregiving in this context [5,23]. Such elevated baseline distress may allow greater observable change following interventions focused on emotional regulation, reflecting heightened unmet support needs at treatment entry and increased receptivity to structured, practical therapeutic input [82]. In addition, observed intervention effects may reflect not only MBCT-specific techniques but also the influence of non-specific therapeutic factors. Participants in the intervention group received additional attention, structured group support, and regular engagement with facilitators and other parents sharing similar caregiving experiences. In constrast, these elements were not provided in the same form to the control group, which continued to receive individual support as usual. As a result, between-group differences may reflect the combined contribution of intervention-specific components and broader contextual and psychosocial factors that were not equally distributed across conditions.

Contemporary neuroscientific models describing the therapeutic mechanisms underlying mindfulness practice provide a coherent explanatory framework for understanding the treatment gains observed in the current study. Research evidence indicates that MBCT induces functional and structural neuroplastic changes in brain system supporting attentional and cognitive control, emotional regulation, and self-referential processing [102]. Indeed, mindfulness practice has been found to enhance activation in prefrontal and anterior cingulate regions, which are critical for top-down emotion regulation [53]. Additionally, it reduces amygdala reactivity, thereby improving threat sensitivity and strengthening emotional resilience [103,104]. In parallel, mindfulness practices modulate the hypothalamic–pituitary–adrenal (HPA) axis, contributing to reductions in cortisol levels [105,106] and counteracting the chronic stress dysregulation frequently reported among parents of children with ASD [107]. As these neurobiological processes unfold, they may support greater interoceptive awareness and regulatory flexibility, facilitating more sustained emotional regulation in the context of chronic caregiving stress.

Because the present study focused on intervening at the individual parent level, the effectiveness of the intervention can be interpreted in relation to broader contextual and cultural factors that may have shaped both treatment engagement and observed outcomes. Importantly, mindfulness-based interventions have not been widely implemented or established as evidence-based approaches within the Greek mental health care system. As a result, participants in the present study were largely unfamiliar with mindfulness practices prior to enrollment. This lack of prior exposure may have influenced both the perceived acceptability of the intervention and the magnitude of observed treatment effects, potentially contributing to substantial improvements from baseline to post-intervention that were maintained at the one-month follow-up. In this context, engagement with mindfulness practices may have represented a novel and meaningful psychological resource, particularly for parents with limited prior access to structured emotional regulation strategies.

In addition, broader socioeconomic conditions may have further shaped parents’ responsiveness to the intervention. The prolonged economic crisis in Greece has disproportionately affected families raising children with autism, who often face increased financial burden related to ongoing therapeutic and educational needs [5,23]. Empirical evidence indicates that these financial pressures are associated with heightened parenting stress, anxiety, and a pervasive sense of insecurity, which may reduce parents’ psychological availability and increase reliance on automatic, reactive parenting responses. Within this context, mindfulness-based interventions that cultivate present-moment awareness and emotional regulation may be particularly salient, offering parents psychological space to disengage from habitual stress-driven reactions. Overall, these contextual factors may have created conditions under which parents were particularly receptive to the intervention, thereby amplifying its perceived effectiveness. At the same time, they underscore the importance of considering how broader contextual influences interact with mindfulness-based interventions to shape acceptability, engagement, and treatment outcomes across diverse settings.

### 4.3. Acceptance of the Intervention

All 30 parents (100%) in the MBCT group completed the eight-week program and consistently attended sessions, engaged in daily mindfulness practice (30–45 min), and applied mindful parenting skills at home. Participants’ consistent involvement throughout the program reflects high commitment and strong feasibility to the intervention protocol. Acceptability ratings were consistently positive with parents reporting that the program’s structure, content, and relevance aligned closely with their needs. They also identified the supportive group environment and the facilitator’s guidance as key strengths of the program. Open-ended responses further revealed the practical value of mindfulness practices for managing everyday parenting challenges. Reported obstacles were primarily associated with increased childcare demands and travel distance to and from the intervention setting. These findings align with international research demonstrating high satisfaction and strong engagement with mindfulness-based programs [73,108,109], confirming MBCT as a feasible and well-accepted intervention for ASD caregivers.

### 4.4. Indirect Parent—Perceived Changes in Children’s Behavior

Although children did not receive direct intervention, parent-reported assessments at post-intervention and follow-up indicated exploratory between-group differences in behavioral outcomes, with the MBCT group showing notable reductions in perceived problem severity. Given that child outcomes were assessed using a single parent-reported item, these findings should be interpreted as parent-perceived changes rather than objective behavioral improvement. Consistent with the transactional model of development [35] and with prior evidence [55,61,110] these parent-perceived gains in children’s behavior may reflect improvements in parents’ emotional regulation and increased use of adaptive parenting strategies. Reductions in parental stress may interrupt maladaptive parent–child transactional cycles and promote a calmer, more supportive caregiving environment that parents may experience as facilitating improvements in their child’s self-regulation. As parents become more regulated, attentive, and responsive to their child’s needs and behaviors, the parent child relationship may improve, and daily interactions may be perceived as less reactive and more supportive. In turn, parents are more able to create a caregiving environment characterized by emotional stability and consistency supporting changes in how child behavior is perceived and managed over time. Therefore, mindful parenting functions as a key mechanism through which parental well-being translates into changes in parents’ perceptions of their child’s behavioral adjustment, underscoring the reciprocal interplay between parental wellbeing and child behavior.

### 4.5. Strengths, Limitations, and Future Research

This study has several methodological strengths. The use of an RCT with an experimental and a waitlist-control group enhances the rigor of the study and supports robust conclusions about the effectiveness of MBCT on parental mental health. Importantly, there was no attrition in the MBCT group, indicating high acceptability, strong engagement, and good feasibility which are all critical factors for interventions targeting parents of children with ASD. Additionally, the inclusion of parents of children aged 4 to 17 years may enhance the applicability of the findings to a broad spectrum of ASD caregiving experiences.

Despite these strengths, several limitations need consideration. First, although the sample size exceeded a priori power criteria, it remained relatively modest, which may limit the precision of effect size estimates and the generalizability of the findings [111]. It should be noted, however, that similar sample sizes are commonly reported in clinical mindfulness-based intervention studies with parents of children with neurodevelopmental conditions, reflecting both the intensity of such interventions and the practical challenges associated with recruiting and retaining this population [38,78,82]. Future studies should recruit larger and more socioeconomically and geographically diverse samples, including families from rural settings and varied cultural backgrounds, to enhance the applicability of results [83]. Second, this study aimed to examine the short-term treatment effects of the MBCT intervention on parental psychopathology; therefore, conclusions regarding the long-term sustainability of treatment gains are limited by the one-month follow-up period [112]. Short-term follow-up assessments (e.g., one to two months) have also been used in pilot and early-stage randomized controlled trials of family mindfulness interventions to examine initial trajectories of change [94,113], as they allow evaluation of early maintenance of effects while minimizing attrition and participant burden before longer-term assessments become feasible. Nevertheless, future research should incorporate extended follow-up assessments spanning several months or years post-intervention in order to more accurately capture long-term trajectories of change. Given that mindfulness is not a stable personality trait but rather a trainable and dynamic capacity, accumulating evidence suggests that the effects of mindfulness-based interventions may not be static, but instead continue to evolve, consolidate, or even emerge more clearly over time [88,91,109].

An additional limitation concerns the absence of an active control condition and the potential influence of expectancy effects. Although the primary aim of the present trial was not to compare MBCT with another active psychological intervention, but rather to evaluate its effects relative to a condition that did not receive MBCT, participants in the control group continued to receive treatment as usual. While this design accounts for ongoing support and routine care [114], as is common in similar studies [38,82,94], improvements in parental mental health and parent-perceived child outcomes in the intervention group may still have been partly influenced by positive treatment expectations, increased attention, or other non-specific factors associated with participation in a structured program. This limitation is particularly relevant given the waitlist control design and the reliance on self-report measures.

Indeed, outcomes in the present study were assessed exclusively using quantitative self-report instruments. Although these measures demonstrate strong psychometric properties [66,70] and are sensitive to change, they may be influenced by cognitive reappraisal processes and positive expectancy effects following participation in a structured intervention. Moreover, self-report measures may not fully capture the subjective complexity of parents’ lived experiences or provide detailed insight into how mindfulness skills are translated into everyday parenting practices. As such, reliance on self-report data may limit the depth with which underlying change processes can be understood.

Finally, child behavioral outcomes in the present study were assessed exclusively through parent-reported ratings, reflecting parents’ perceptions of changes in their child’s behavior over time. Although parent reports provide ecologically valid information about daily functioning, this approach may be influenced by expectancy effects and reporting biases, particularly in the context of psychosocial interventions targeting parental emotional regulation [115]. In addition, reliance on a single informant does not allow differentiation between perceived behavioral change and objective behavioral improvement, nor does it adequately account for the presence of co-occurring psychopathology (e.g., attention-deficit/hyperactivity disorder), which may independently shape children’s behavioral presentations and trajectories [116]. Consequently, findings related to child behavior should be interpreted as preliminary and parent-perceived changes rather than definitive evidence of direct behavioral improvement. Future research would benefit from incorporating multi-informant assessment strategies (e.g., reports from teachers and clinicians), structured behavioral observations, and standardized assessment tools to enhance the validity and robustness of child outcome evaluation.

## 5. Conclusions

This RCT provides robust evidence that MBCT, combined with mindful parenting practices, is an effective and acceptable intervention for parents of children with ASD. Parents who received MBCT demonstrated significant improvements in overall mental health and wellbeing, with particularly notable reductions in parental psychopathology along with strong gains in positive affect and life satisfaction. These improvements were evident at post-intervention and either further increased or, in the case of anxiety, remained stable at follow-up, revealing both the direct impact and short-term durability of treatment gains. In contrast, the waitlist-control group showed no meaningful changes, underscoring both the efficacy of MBCT and the persistent psychological burden experienced by parents caring for a child with ASD.

Preliminary indications of parent-perceived changes in children’s behavior suggest that improvements in parental emotional regulation may positively influence family dynamics and parent–child relationships. These improvements are likely explained by gains in present-moment awareness, acceptance, and self-compassion all of which are central mechanisms cultivated through MBCT and mindful parenting. As a result, through these practices parents experience neurocognitive changes which interrupt maladaptive cognitive patterns and reduce emotional reactivity while enhancing prefrontal control processes involved in psychological flexibility and emotional regulation. Thus, these changes enable parents to respond to caregiving demands in a more adaptive and flexible manner rather than relying on habitual stress or emotional-driven reactions [6,117].

In clinical practice, MBCT represents a well-accepted intervention that can be integrated into individual therapy, group-based parent support, and broader psychoeducational programs. By strengthening parental mental health, MBCT has the potential to indirectly support children’s positive behavioral, highlighting its value as a family-centered approach to promoting psychological well-being and healthier parent–child interactions.

## Data Availability

Data is available upon reasonable request from the corresponding author.
